# Tonographic Effect of Ocular Response Analyzer in Comparison to Goldmann Applanation Tonometry

**DOI:** 10.1371/journal.pone.0169438

**Published:** 2017-01-09

**Authors:** Martin Zimmermann, Susanne Pitz, Irene Schmidtmann, Norbert Pfeiffer, Joanna Wasielica-Poslednik

**Affiliations:** 1 Department of Ophthalmology, University Medical Center of the Johannes Gutenberg-University Mainz, Mainz, Germany; 2 Institute for Medical Biostatistics, Epidemiology and Informatics (IMBEI), University Medical Center of the Johannes Gutenberg-University Mainz, Mainz, Germany; Bascom Palmer Eye Institute, UNITED STATES

## Abstract

**Aims:**

The tonographic effect is a phenomenon of intraocular pressure (IOP) reduction following repeated tonometry. This study examines whether the tonographic effect occurs following IOP measurement performed with Ocular Response Analyzer (ORA).

**Methods:**

Both eyes of 31 glaucoma patients and 35 healthy controls underwent nine IOP-measurements performed with GAT and ORA. The number of GAT and ORA measurements performed on each eye differed depending on the randomly allocated investigation scheme. Central corneal thickness (CCT), anterior chamber volume (ACV) and anterior chamber depth (ACD) were assessed with Pentacam before and after the repeated GAT/ORA measurements.

**Results:**

There was no statistically significant tonographic effect for IOP readings obtained by the ORA: corneal compensated intraocular pressure (IOPcc) (-0.11 ± 3.06 mmHg, p = 0.843 in patients and -0.71 ± 3.28 mmHg, p = 0.208 for controls) and Goldmann-correlated intraocular pressure (IOPg) (-0.31 ± 2.38 mmHg, p = 0.469 in patients and -0.31 ± 2.37 mmHg, p = 0.441 in controls) measured with ORA. There was a significant IOP reduction from the first to the second GAT measurement, i.e. tonographic effect (-0.55 ± 2.00 mmHg, p = 0.138 in patients and -1.15 ± 1.52 mmHg, p < 0.001 in controls). CCT, corneal hysteresis (CH) and corneal resistance factor (CRF) were lower in glaucoma patients. The repeated IOP measurements resulted in an increase of CCT in all subjects (but no change of ACV and ACD). The tonographic effect of GAT correlated with CCT in glaucoma patients (r = 0.37).

**Conclusion:**

In contrast to GAT, repeated ORA measurements do not result in the tonographic effect. Repeated IOP measurements resulted in an increase of central corneal thickness, but did not influence the volume and depth of anterior chamber.

## Introduction

The measurement of intraocular pressure (IOP) is an important means to detect and manage glaucoma [1, 2]. The gold standard for IOP measurement still is Goldmann applanation tonometry (GAT). There are multiple sources of error that may influence the accuracy of GAT measurements [3, 4]. One of the known phenomena is the reduction of the IOP through repeated applanation, also called “tonographic effect” [5–11]. Stocker [5] mentions the amount of aqueous humor pressed out of the anterior chamber as one of the reasons for the IOP drop. However, he hypothesized that the mechanic process of forcing out the aqueous humor alone cannot explain the tonographic phenomenon, but suspected a reflectory change of aqueous formation as a further variable. Moses [6] suspected topical anesthetics as being relevant for the tonographic effect.

In order to increase the measurement reliability in clinical trials, a mean value of several repeated IOP-measurements is used [12–15]. Likewise, young ophthalmologists or optometrists at the beginning of their learning curve often need more tries to measure IOP properly. However, repeated GAT measurements bear the risk of false results through the tonographic effect.

Ocular Response Analyzer (ORA) is an IOP measurement technique, which utilizes a visco-elastic structure of the human corneal tissue in a non-contact way. ORA provides both: IOP and biomechanical properties of the cornea such as corneal hysteresis (CH) and corneal resistance factor (CRF). A corneal-compensated intraocular pressure (IOPcc) has been reported to be less affected by the corneal properties than GAT [16–18]. Furthermore, CH and CRF seem to be predictors for the development of glaucoma [19]. The lack of contact with the cornea eliminates the need for topical anesthetics and reduces the risk of cross-infection and corneal damage [20–22].

The ORA-measurements have shown to be reproducible in several studies [17, 18, 23]. There are only few studies evaluating an influence of repeated ORA measurements or ocular massage on the IOP in healthy subjects [17, 24, 25].

The purpose of our prospective, randomized clinical trial was to compare ORA and GAT regarding the tonographic effect in glaucoma patients and in healthy controls. Furthermore, we wanted to evaluate the possible correlation between the tonographic effect and parameters as central corneal thickness (CCT), anterior chamber volume (ACV), anterior chamber depth (ACD), CH, and CRF.

## Materials and Methods

This prospective, randomized, single-center, open, controlled study with two parallel study groups was carried out in accordance with the Declaration of Helsinki. Ethics approval was obtained from the Ethics committee of Rhineland-Palatinate, Germany.

Glaucoma patients were recruited from the glaucoma section of the Department of Ophthalmology of the University Medical Center of the Johannes Gutenberg-University Mainz. In order to obtain similar study groups, sex- and age-matched healthy controls were recruited from the staff of the University Medical Center of Mainz and from the patients of a family practice in Mainz, Germany. After a detailed personal survey conversation, a written informed consent was obtained from all study participants. All subjects were then evaluated at the Department of Ophthalmology of the University Medical Center of the Johannes Gutenberg-University Mainz between December 2013 and March 2015. Inclusion criteria for both groups were: female or male of any race aged 18 years or older, ability to understand the character and individual consequences of the clinical trial, signed and dated informed consent must have been available before the start of any specific trial procedure; in the control group: normal ophthalmological status, no glaucoma or other relevant eye diseases, IOP ≤ 21 mmHg; in the patients´ group: diagnosis of primary open-angle glaucoma (POAG), pseudoexfoliation glaucoma (PEXG) or pigment dispersion glaucoma (PG) with characteristic alterations of the optic nerve head and correlating visual field deficiencies.

The exclusion criteria in both groups were: astigmatism > 2.0 diopters; spherical refraction > 3.0 diopters; CCT < 500 μm; CCT > 600 μm; pseudophakia; corneal, conjunctival, or intraocular inflammatory eye disease; use of contact lenses within three months before study examination; any corneal pathologic condition; history of previous ocular surgery.

### Study procedure

All subjects underwent an assessment of an objective refraction, the best corrected visual acuity (BCVA) with Snellen charts, a slit lamp examination and indirect ophthalmoscopy in miosis.

Each eye underwent nine measurements of the IOP, with both GAT and ORA. The number of IOP measurements differed according to the examination scheme that was assigned in a randomized order to each patient or healthy control (Table 1). For instance, in scheme 1, the left eye (OS) was measured first with triple ORA, triple GAT, and then triple ORA again. Afterwards, the right eye (OD) was measured with triple GAT, triple ORA, and then triple GAT. The time lapse between measurements with different devices was kept as short as possible, i.e. between 25–130 seconds, depending on the mobility of the patients or individual positioning difficulties.

**Table 1:** Four examination schemes for IOP measurements.

Scheme	1st Eye	Period 1	Period 2	Period 3	2nd Eye	Period 1	Period 2	Period 3
**1**	OS	ORA	GAT	ORA	OD	GAT	ORA	GAT
**2**	OS	GAT	ORA	GAT	OD	ORA	GAT	ORA
**3**	OD	ORA	GAT	ORA	OS	GAT	ORA	GAT
**4**	OD	GAT	ORA	GAT	OS	ORA	GAT	ORA

ORA, Ocular Response Analyzer; GAT, Goldmann applanation tonometry; OD, right eyes; OS, left eyes.

GAT was performed by one experienced ophthalmologist and the same calibrated tonometer was used through the whole study. ORA- and Pentacam measurements were performed by a medical student, who was experienced and trained in handling of these devices. The measurement of IOP with GAT required instilling oxybuprocaine-HCl/fluorescein-Na (Thilorbin®, OmniVision) eye drops in the lower conjunctival cul-de-sac in both eyes and was performed without pupil dilatation in all cases. The application of one anesthetizing eye drop was performed once per eye, regardless of whether the eye was measured with GAT once or twice. Each GAT measurement was performed three times and the mean value was taken for the statistical analysis.

The ORA (Reichert Inc., Depew, USA, software version 2.0) utilizes a visco-elastic structure of the human corneal tissue in a dynamic bi-directional applanation process. The difference in inward and outward pressure values is called CH and the average of both values provides Goldmann-correlated intraocular pressure (IOPg). Calculated on the basis of the measured CH, the ORA provides two other parameters: CRF and IOPcc. According to our study protocol, three measurements were performed and the value with the best wave-score was taken for the statistical analysis.

The Pentacam (Oculus Pentacam HR Typ 70900, Oculus, Weimar, Germany) is a device combining a slit illumination and Scheimpflug camera, which rotate together around the eye. The CCT, ACD and ACV were assessed before and after the IOP session. The average of three values was taken for further analysis.

### Statistical analysis

The sample size was chosen such that six pairwise t-tests could be performed on a 5% significance level, controlling for multiple testing by using a Bonferroni correction, i.e. a significance level of 0.83% for six pairwise t-tests. A power of 81% was calculated for 70 study participants in order to be able to detect an IOP difference of 1 mmHg with a standard deviation of 3 mmHg. The statistical analysis was performed using Microsoft Excel 2007, IBM SPSS Statistics Version 20, and SAS 9.4. First, descriptive were obtained, i.e. absolute and relative frequencies for categorical variables, mean, standard deviation, minimum, maximum, median and quartiles for quantitative variables. Second, six confirmatory t-tests were performed to identify differences between measurements taken before and after ORA and GAT and between glaucoma patients and probands. The results of these confirmatory t-tests were complemented with a three-period crossover analysis employing a linear model. Moreover, several exploratory t-tests concerning IOP and corneal differences between glaucoma patients and probands were performed.

The Pearson correlation coefficient was used to assess correlations between IOP values (GAT-IOP, IOPg, IOPcc), corneal parameters (CH, CRF), and Pentacam measurements (CCT, ACV and ACD). A correlation coefficient ≥ 0.7 was considered a strong correlation; ≥ 0.5 was considered a moderate correlation; and < 0.5 a weak correlation.

## Results

Both eyes of 31 glaucoma patients (21 female, aged 63.3 ± 8.2 years, range = 40–75 years) and sex- and age-matched 35 healthy probands (21 female, aged 65.2 ± 9.2 years, range = 44–84 years) were enrolled in this study. Originally, 35 patients were included. However, four patients were excluded after measurement values did not meet the inclusion criteria. Thirty patients were diagnosed with primary open angle glaucoma (POAG), while one patient was diagnosed with pseudoexfoliation glaucoma (PEXG). All patients and probands were phakic. All glaucoma patients were treated with topical antiglaucoma agents.

### IOP values

Concerning mean GAT-IOP, IOPg, IOPcc, no differences between patients and healthy controls were identified (Table 2). In the case of multiple measurement sessions for the same eye, the mean of these sessions was employed.

**Table 2:** Mean IOP values ± standard deviation.

	GAT-IOP OD	GAT-IOP OS	IOPcc OD	IOPcc OS	IOPg OD	IOPg OS
Patients	13.86 ± 3.39	13.47 ± 3.19	16.23 ± 4.35	16.43 ± 4.18	14.32 ± 4.44	15.16 ± 5.01
Probands	13.90 ± 3.44	14.71 ± 2.66	16.40 ± 4.96	16.14 ± 4.53	15.95 ± 4.29	16.58 ± 4.56
p-value	0.960	0.091	0.881	0.788	0.134	0.231

GAT-IOP, Goldmann applanation tonometry; IOPcc, corneal-compensated IOP; IOPg, Goldmann-correlated IOP; OD, right eyes; OS, left eyes; in mmHg.

GAT-IOP was systematically lower than IOPcc: mean differences between IOPcc and GAT-IOP of 2.44 ± 3.07 mmHg for OD (p < 0.001) and 2.15 ± 3.37 mmHg for OS (p < 0.001) were found.

IOPcc, IOPg and GAT showed no differences between the right and the left eyes.

### Tonographic effect

**Fig 1:**
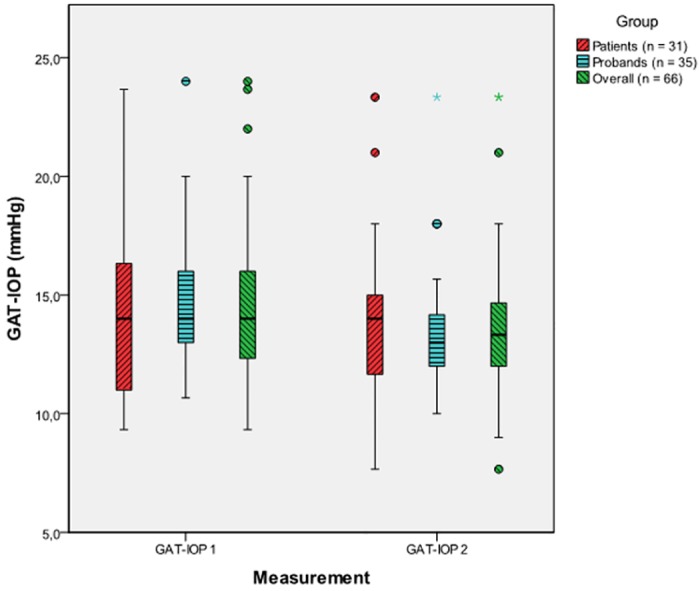
Tonographic effect from the first (GAT-IOP 1) to the second (GAT-IOP 2) GAT measurement for patients, probands and the overall sample (patients and probands combined), [mmHg].

The significant mean IOP decrease from the first to the second GAT measurement was -0.87 ± 1.77 mmHg, p < 0.001 in the same eye for patients and probands. This was also confirmed by the results of the linear model. The average difference between the first and the second mean GAT-IOP was -0.55 ± 2.00 mmHg (p = 0.138) for the glaucoma patients and -1.15 ± 1.52 mmHg (p < 0.001) for controls (Fig 1). No significant difference between glaucoma patients and healthy probands concerning the magnitude of the tonographic effect could be found (p = 0.196).

**Fig 2:**
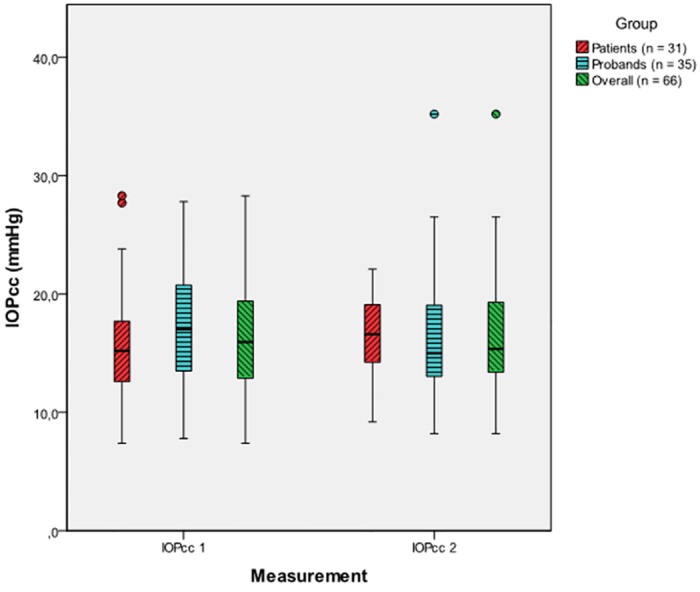
Tonographic effect from the first (IOPcc 1) to the second (IOPcc 2) IOPcc measurement for patients, probands and the overall sample (patients and probands combined), [mmHg].

The mean difference between the first and the second IOPcc for both study groups combined was -0.43 ± 3.17 mmHg (p = 0.276), thus being non-significant. This was also confirmed by the results of the linear model. The average difference between the first and the second IOPcc was -0.11 ± 3.06 mmHg in patients (p = 0.843) and -0.71 ± 3.28 mmHg for controls (p = 0.208), no significant difference between patients and probands could be found (p = 0.446) (Fig 2).

**Fig 3:**
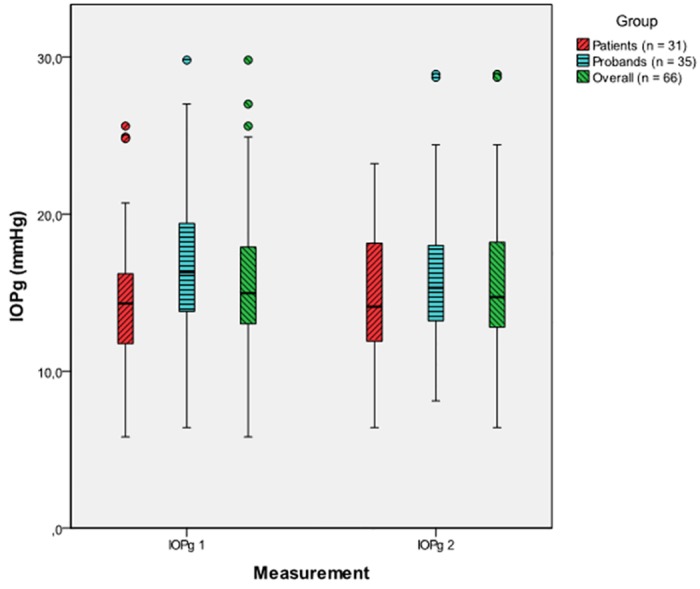
Tonographic effect from the first (IOPg 1) to the second (IOPg 2) IOPg measurement for patients, probands and the overall sample (patients and probands combined), [mmHg].

The mean difference between the first and the second IOPg was -0.31.± 2.35 mmHg, (p = 0.285) for both groups combined (non-significant). This was also confirmed by the results of the linear model. The average difference between the first and the second IOPg was -0.31± 2.38 mmHg (p = 0.469) in glaucoma patients and -0.31 ± 2.37 mmHg (p = 0.441) in controls. There was no significant difference between patients and probands (p = 0.998) (Fig 3).

### Pentacam parameters

Mean CCT was noticeably thinner in glaucoma patients in comparison to healthy controls in both measurements in both eyes (Table 3).

**Table 3:** Mean values and standard deviations of central corneal thickness before and after IOP measurements for patients and probands.

	CCT 1_OD	CCT 1_OS	CCT 2_OD	CCT 2_OS
Patients	538.46 ± 19.65	535.74 ± 20.03	541.05 ± 20.31	541.63 ± 18.99
Probands	556.20 ± 24.34	557.56 ± 24.49	564.48 ± 25.44	562.02 ± 26.48
p-value	0.002	< 0.001	< 0.001	0.001

CCT, central corneal thickness; CCT 1, CCT before IOP measurements; CCT 2, CCT after IOP measurements; OD, right eyes; OS, left eyes; μm.

The repeated GAT/ORA IOP measurements led to a relevant increase of CCT in all subjects (Table 4).

**Table 4:** Mean values and standard deviations for differences between first and second CCT measurements.

	CCT1_2_OD	CCT1_2_OS
Value ± SD	5.61 ± 9.41	5.13 ± 7.39
p-value	< 0.001	< 0.001

SD, standard deviation; CCT1_2, mean values and SDs for differences between first and second CCT measurements; OD, right eyes; OS, left eyes; μm.

ACV and ACD did not differ between patients and probands (Table 5). The repeated IOP-measurements did not influence the ACD in both eyes and ACV in the right eyes. Only the ACV in the left eyes showed a decrease between the first and the second Pentacam measurement (p = 0.02).

**Table 5:** Anterior chamber volume and anterior chamber depth before and after repeated GAT/ORA IOP measurements for patients and probands for the right and the left eyes.

	ACV 1_OD	ACV 1_OS	ACV 2_OD	ACV 2_OS	ACD 1_OD	ACD 1_OS	ACD 2_OD	ACD 2_OS
Patients	142.65 ± 32.67	140.18 ± 33.00	141.59 ± 32.73	140.02 ± 33.43	2.66 ± 0.33	2.67 ± 0.31	2.65 ± 0.33	2.66 ± 0.33
Probands	129.60 ± 34.54	132.74 ± 33.42	128.74 ± 32.37	130.55 ± 31.08	2.55 ± 0.39	2.57 ± 0.38	2.55 ± 0.38	2.57 ± 0.38
p-value	0.121	0.367	0.114	0.238	0.241	0.255	0.265	0.325

ACV, anterior chamber volume [mm^3^]; ACD, anterior chamber depth [mm]; ACV 1/ACD 1, values before repeated GAT/ORA measurements; ACV 2/ACD 2, values after repeated GAT/ORA IOP measurements; OD, right eyes; OS, left eyes.

### Crossover model

The confirmatory t-tests verify the results of the three period crossover analyses, with GAT values on average at 1.98 mmHg (p = 0.0028) lower than IOPcc values (95% CI: [-3.26, -0.69]). When comparing IOPcc (ORA) with GAT we found no carry-over effects (p = 0.29), we also found no period effect (p = 0.29). However, we found a significant treatment effect. Therefore, still no tonographic effect could be observed for the ORA measurements. No difference was found for a right versus left eye comparison.

## Discussion

In order to find out whether the tonographic effect concerns ORA measurements, we employed a cross-over analysis with multiple GAT and ORA IOP-measurements randomly alternating between the right and the left eyes of healthy controls and glaucoma patients. In contrast to GAT, we did not find any significant decrease of IOPcc and IOPg between the first and the second ORA measurement and therefore no statistically or clinically significant tonographic effect in either glaucoma patients or healthy controls. Only two studies report about the effect of repeated ORA measurements on IOP: Goebels et al. [24] found IOPcc and IOPg values decreasing by -1.05 and -1.19 mmHg within a sequence of five sequential ORA readings in healthy subjects. This effect was more pronounced than in the healthy population of our study (-0.71 ± 3.28 mmHg, p = 0.208 for IOPcc and -0.31 ± 2.37 mmHg, p = 0.441, for IOPg). This may be explained by a higher number of single ORA measurements (5 sets with 4 readings each) or by the younger age of their population (mean 35 years, range 17–59 years) compared to the age of our healthy population (65.2 years, range 44–84 years). Corneal stiffness was shown to increase with age [26–32]. However, the authors do not mention any significances or the time intervals between the ORA measurements, thus making comparisons difficult. David et al. [17] compared 12 readings (3 sets with 4 readings each) of ORA parameters (IOPcc, IOPg, CH, and CRF) obtained within a 30-minute period in healthy subjects. They found significantly higher IOPcc and IOPg values in the first set of measurements compared to later readings with a maximum difference of -0.52 mmHg (p = 0.01) for IOPcc and -0.49 mmHg (p = 0.004) for IOPg (difference between sessions one and two for right eyes). Lam and Chen [25] evaluated the effect of ocular massage on ORA parameters in young healthy subjects, finding a significant decrease of IOPcc of about 2.8 mmHg. As important ameliorations, our study compared ORA results with GAT and took wave score as a quality criterion into consideration. A purpose-built additional three period cross-over analysis helped to detect any tonographic effects. Moreover, to the best of our knowledge our study examined the tonographic effect of ORA for the first time in glaucoma patients and in age-matched controls.

There have been only few attempts to determine the tonographic effect for other non-contact tonometers [11, 33]. Al-Mubrad and Ogbuehi [34] examined the tonographic effect of Keeler Pulsair EasyEye and Topcon CT80 in 120 healthy subjects by using a randomized examination scheme with different combinations of CT80, Keeler and GAT, measuring three times (Keeler, GAT) or four times (CT80). They found an IOP-decrease of -1.0 ± 2.3 mmHg (p < 0.05) for Keeler Pulsair EasyEye and -0.6 ± 1.7 mmHg (p < 0.05) for Topcon CT80 in healthy subjects and therefore a statistically significant tonographic effect.

GAT-IOP was systematically lower in comparison to IOPcc in our study. Similar results were found by several researchers [12, 14, 35–41]. With regard to mean GAT-IOP, IOPcc and IOPg values, no differences could be found between patients and healthy probands. The fact that all glaucoma patients were using IOP-lowering medications at the time of the study might explain the lack of IOP differences between patients and probands. The phenomenon of similar IOP values between glaucoma patients under therapy and probands can be observed in other studies such as these of Costin et al. [42] or Sullivan-Mee et al. [19].

In our study we found a statistically significant tonographic effect of GAT. We recorded the GAT-IOP reduction of mean -0.55 ± 2.00 mmHg for glaucoma patients and -1.15 ± 1.52 mmHg for healthy controls. The more pronounced tonographic effect in healthy controls in comparison to glaucoma patients may be explained by a pathologically changed trabecular meshwork of the glaucomatous eyes [43, 44] The time gap between the two GAT measurements was approximately three minutes. Since Stocker [5] there have been numerous attempts to analyze the tonographic effect of GAT (Table 6). The study applying a quite similar study design concerning GAT is that of Recep et al. [10], who obtained similar GAT-IOP differences after three minutes in healthy subjects after employing oxybuprocaine and fluorescein for GAT-IOP measurement. Our results are also comparable with other mentioned studies. However, they either measured only normal subjects or only glaucomatous patients [5–8, 45–47] or only glaucomatous patients [9], or do not give any information about the significance of their results. [8, 45, 47]. The other works employed a slightly different setting with very short time gaps between GAT-IOP measurements [33, 48, 49]. Overall, despite the statistical significance of the tonographic effect for GAT-IOP, an IOP change in the range of -0.5 to -1.15 mmHg does not seem to have decisive relevance in a clinical setting.

**Table 6:** Overview of the studies analyzing the tonographic effect of contact and non-contact tonometers.

Author(s)	Year	IOP/IOP change	Sig. (p)	Setup	Sample (n)	Eye status	Device
Stocker [5]	1958	-0.97 mmHg	n.a.	30s after first reading on same eye	20	normal	Schiötz tonometer
		-1.90 mmHg	n.a.	4 mins after first reading on same eye	20	normal	
		-2.72 mmHg	n.a.	4 mins after the tonometer resting on other eye	20	normal	
		-2.37 mmHg	n.a.	on healthy eyes	12	normal	
		-3.25 mmHg	n.a.	on glaucomatous eyes	8	glaucomatous	
Armaly & Rubin [37]	1961	-0.26 ± 0.75 to -0.39 ± 0.81 mmHg	sig.	GAT values after GAT once a minute for six minutes	20	normal	GAT
Moses [6]	1961	-0.9 mmHg*	n.a.	1 min after first reading on right eye	25	normal	GAT
		+0.1 mmHg*	n.a.	2 mins after first reading on right eye	25		
		-1 mmHg*	n.a.	3 mins after first reading on right eye	25		
		-0.05 mmHg*	n.a.	4 mins after first reading on right eye	25		
		-0.9 mmHg*	n.a.	5 mins after first reading on right eye	25		
		-0.1 mmHg*	n.a.	1 min after first reading on left eye, 7 mins after first reading on right eye	25		
		+0.1 mmHg*	n.a.	2 mins after first reading on left eye, 8 mins after first reading on right eye	25		
		-0.5 mmHg*	n.a.	3 mins after first reading on left eye, 9 mins after first reading on right eye	25		
		-0.4 mmHg*	n.a.	4 mins after first reading on left eye, 10 mins after first reading on right eye	25		
		-0.1 mmHg*	n.a.	5 mins after first reading on left eye, 11 mins after first reading on right eye	25		
Bechrakis [9]	1966	-3.70 mmHg	n.a.	4 mins after first reading on same eye	17	glaucomatous	GAT
		-5.7 ± 1.3 mmHg	n.a.	12 mins after first reading on same eye	17		
		-5.1 mmHg	n.a.	12 mins after first reading on same eye	17		
		-3.58 ± 1.2 mmHg	n.a.	4 mins after first reading on same eye	44		
Moses & Liu [39]	1968	-0.40 ± 1.40 mmHg	< 0.05	right eye, rising and approx. 30 feet of walking between measurements	74	glaucomatous	GAT
		-0.16 ± 1.43 mmHg	n.a.	left eye, rising and approx. 30 feet of walking between measurements	74		
Krakau & Wilke [7]	1971	-2.9 mmHg	n.a.	5 mins after the first measurement and one measurement each minute on the same eye	16	normal	GAT
		-4.6 mmHg	n.a.	5 mins after the first measurement and one measurement each minute on the same eye	5		
Wilke [8]	1972	-0.4 mmHg	> 0.10	A) 5 mins on the same eye after measurement on both eyes	6	normal	GAT
		-2.6 mmHg	< 0.001	B) 5 minutes on the same eye after measurement once a minute for 5 minutes	6		
		-3.2 mmHg	< 0.001	C) Patients seated for 5 mins after having local anesthesia, then measurements as in B)	6		
		-2.9 mmHg	< 0.001	Sham measurements without contact to cornea, then as in B)	6		
Phelps & Phelps [36]	1976	-0.4 ± 2.6 mmHg	< 0.05	Mean IOP change in the right eye after 15 (5–30 minutes range) minutes of walking	210	normal	GAT
		-0.2 ± 2.4 mmHg	> 0.05	Mean IOP change in the left eye after 15 (5–30 minutes range) minutes of walking	210		
Thorburn [38]	1979	-0.5 ± 0.9 mmHg	n.a.	IOP change with same investigator, time interval unclear	20	in-patients	GAT
		-0.5 ± 0.9 mmHg	n.a.	IOP change with two different investigators for 1st and 2nd measurement, time interval unclear	72		
Motolko et al.	1982	18.81 mmHg		Mean of first three GAT measurements in 30s intervals	9	normal	GAT
[35]		17.48 mmHg	< 0.05	Mean of fourth through sixth GAT mesaurement in 30s intervals	9		
		16.78 mmHg	< 0.05	Mean of seventh through ninth GAT measurement in 30s intervals	9		
Recep et al. [10]	1998	-1.33 ± 1.86 mmHg	< 0.05	1 min on the same eye after first measurement	34	normal	GAT
		-0.53 ± 1.78 mmHg	> 0.05	2 mins on the same eye after first measurement	29		
		-1.89 ± 1.57 mmHg	< 0.05	3 mins on the same eye after first measurement	27		
		-2.29 ± 1.07 mmHg	< 0.05	4 mins on the same eye after first measurement	41		
		-1.92 ± 2.08 mmHg	< 0.05	5 mins on the same eye after first measurement	33		
		-0.00 ± 1.17 mmHg	> 0.05	10 mins on the same eye after first measurement	28		
Stewart et al. [40]	2004	IOP 1) 24.9 ± 2.7, IOP 2) 25.0 ± 2.8, IOP 3) 24.9 ± 2.8 mmHg	> 0.05	3 IOP readings of untreated baseline IOPs within a few seconds at 8 am	33	glaucomatous	GAT
		IOP 1) 16.3 ± 2.9, IOP 2)16.1 ± 3.0, IOP 3) 16.3 ± 2.9 mmHg	> 0.05	3 IOP readings of treated final IOPs within a few seconds at 8 am	33		
Lam et al. [25]	2007	1) 15.32 ± 2.30 2) 12.56 ± 3.08	sig.	IOPcc before (1) and after (2) ocular massage for 5 mins	53	normal	ORA
		1) 11.03 ± 1.31 2) 11.63± 1.46	sig.	CH before (1) and after (2) ocular massage for 5 mins	53		
		1) 10.96 ± 1.60 2) 10.72 ± 1.65	sig.	CRF before (1) and after (2) ocular massage for 5 mins	53		
		> +1.00 mmHg	n.a.	Distribution of change in IOPcc from ocular massage	2		
		+1.00 to -1.00 mmHg	n.a.	Distribution of change in IOPcc from ocular massage	11		
		-1.01 to -3.00 mmHg	n.a.	Distribution of change in IOPcc from ocular massage	14		
		-3.01 to -5.00 mmHg	n.a.	Distribution of change in IOPcc from ocular massage	18		
		> -5.00 mmHg	n.a.	Distribution of change in IOPcc from ocular massage	8		
AlMubrad & Ogbuehi [23]	2010	1.0 ± 2.3 mmHg	< 0.05	IOP difference before and after GAT, min. 48 hrs between sessions, interval between GAT and non-contact tonometry 2–5 mins	60	normal	Keeler Pulsair EasyEye
		0.6 ± 1.7 mmHg	< 0.05	IOP difference before and after GAT, min. 48 hrs between sessions, interval between GAT and non-contact tonometry 2–5 mins	60		Topcon CT80
Gaton et al. [26]	2010	IOP 1) 15.94 ± 4.3 mmHg	< 0.0001	GAT readings in interval of few seconds	67	glaucomatous	GAT
		IOP 2) 14.90 ± 4.5 mmHg			67		
		IOP 1) 13.64 ± 3.8 mmHg	0.83	GAT readings in interval of few seconds	70	normal	
		IOP 2) 13.73 ± 3.2 mmHg			70		
Goebels et al. [24]	2012	14.92 mmHg	-	Mean IOPcc measurement 1	45	normal	ORA
		14.14 mmHg	-	Mean IOPcc measurement 2	45		
		13.78 mmHg	-	Mean IOPcc measurement 3	45		
		13.91 mmHg	-	Mean IOPcc measurement 4	45		
		13.87 mmHg	-	Mean IOPcc measurement 5	45		
		15.72 mmHg	-	Mean IOPg measurement 1	45		
		14.92 mmHg	-	Mean IOPg measurement 2	45		
		14.49 mmHg	-	Mean IOPg measurement 3	45		
		14.58 mmHg	-	Mean IOPg measurement 4	45		
		14.53 mmHg	-	Mean IOPg measurement 5	45		
David et al. [17]	2013	-0.493 mmHg	0.004	IOPg difference between sessions 1 and 2, right eyes	100	normal	ORA
		-0.241 mmHg	0.08	IOPg difference between sessions 1 and 2, left eyes	100		
		-0.521 mmHg	0.01	IOPcc difference between sessions 1 and 2, right eyes	100		
		-0.269 mmHg	0.11	IOPcc difference between sessions 1 and 2, left eyes	100		
		0.011 mmHg	0.93	IOPg difference between sessions 2 and 3, right eyes	100		
		-0.168 mmHg	0.10	IOPg difference between sessions 2 and 3, left eyes	100		
		0.141 mmHg	0.43	IOPcc difference between sessions 2 and 3, right eyes	100		
		-0.148 mmHg	0.35	IOPcc difference between sessions 2 and 3, left eyes	100		
		-0.482 mmHg	0.002	IOPg difference between sessions 1 and 3, right eyes	100		
		-0.409 mmHg	0.008	IOPg difference between sessions 1 and 3, left eyes	100		
		-0.380 mmHg	0.03	IOPcc difference between sessions 1 and 3, right eyes	100		
		-0.417 mmHg	0.02	IOPcc difference between sessions 1 and 3, left eyes	100		

* IOP estimations as observed in graphic.

Another aspect of this study was to analyze whether a correlation between corneal parameters and the tonographic effect exists. We identified a weak (r = 0.37), but mentionable (p = 0.042) correlation between the tonographic effect of GAT and CCT. This was true only for patients, but not for the healthy controls (r = -0.107). To the best of our knowledge, we executed the only study that evaluated the relationship of CCT with the GAT-IOP change, i.e. the tonographic effect for glaucomatous eyes. We can speculate, the higher the initial CCT, the higher the tonographic effect in glaucoma patients or vice versa. Baseline CCT was thinner in glaucoma patients in comparison to healthy controls. Similar effects were found by other authors either [34, 41, 42, 46, 47–50].

Repeated IOP-measurements resulted in a noticeable increase of CCT, but no change of the anterior chamber depth and volume. Ocular massage through applanation tonometry might cause damage to the corneal epithelium and thus soften structures and/or increase penetration of anesthetics [3, 22]. This might be crucial for a CCT change as well: Al-Mubrad and Ogbuehi [20], p.307, mention a study by Weekers [50], stating “that topical anesthetics caused an alteration of the endothelial Na+/K+ pump resulting increased stromal hydration and as a consequence, increased corneal thickness.” Herse and Siu [51] also suspect the appearance of transitory edema of the corneal stroma as a cause for the CCT increase. Rosa et al. [52] mention the preservatives present in the ophthalmic solutions as a trigger for structural/functional damage to both the corneal epithelium and endothelium, thus increasing CCT in other studies. The increased corneal penetration caused by applanation tonometry could explain why a CCT increase with potential edema etc. was observed without any change in ACV or ACD. Moreover, since the restoration of ACV presumably takes between 2–5 minutes, it is very likely that any ACV reduction was compensated for before the final Pentacam measurements [11, 53].

The study has certain limitations: concerning participating glaucoma patients, an inhomogeneous group with supposedly different kinds and numbers of topical medication were included. The differences in medication might have differing effects on corneal properties etc. As a further consequence, only glaucoma patients with normal IOP values were included in the study. Thus, conclusions cannot be drawn for (untreated) glaucoma patients with elevated IOP values.
